# Methylated Septin 9 as a Promising Biomarker in the Diagnosis and Recurrence Monitoring of Colorectal Cancer

**DOI:** 10.1155/2022/7087885

**Published:** 2022-07-02

**Authors:** Pingxia Lu, Xianjin Zhu, Yanfang Song, Yue Luo, Junsheng Lin, Junrong Zhang, Yingping Cao, Zhengyuan Huang

**Affiliations:** ^1^Department of Laboratory Medicine, Fujian Medical University Union Hospital, 29 Xinquan Road, Fuzhou 350001, China; ^2^Clinical Laboratory, Department of Laboratory Medicine, The Affiliated People's Hospital of Fujian University of Traditional Chinese Medicine, 602 Bayiqi Road, Fuzhou 350001, China; ^3^Department of Clinical Laboratory, School of Medical Technology and Engineering, 88 Jiaotong Road, Fuzhou, 350004 Fujian, China; ^4^Department of Emergency Surgery, Fujian Medical University Union Hospital, 29 Xinquan Road, Fuzhou 350001, China

## Abstract

**Purpose:**

The clinical utility of plasma methylated septin 9 (*mSEPT9*) DNA in screening and recurrence monitoring for colorectal cancer (CRC) is highly promising. The present study was performed to determine the diagnostic value of *mSEPT9* in CRC detection and recurrence monitoring in Chinese patients.

**Methods:**

Overall, 616 patients newly diagnosed with CRC and 122 individuals with no evidence of disease were recruited from October 1, 2019, to May 31, 2021, at Fujian Medical University Union Hospital. Plasma and serum samples were collected for analyzing *mSEPT9*, carcinoembryonic antigen (CEA), and carbohydrate antigen-19-9 (CA19-9). Data on clinicopathological characteristics were collected and analyzed. Sensitivity and specificity were calculated to evaluate the diagnostic potential of each marker; the receiver operating characteristic (ROC) curve was applied for the assessment of diagnostic value, and comparisons among *mSEPT9*, CEA, CA19-9, and their combination were assessed via ROC curves.

**Results:**

*mSEPT9* achieved an overall sensitivity and specificity of 72.94% and 81.97%, respectively, with an area under the curve (AUC) value of 0.826, which were higher than those of CEA (sensitivity: 43.96%; specificity: 96.72%; AUC: 0.789) and CA19-9 (sensitivity: 14.99%; specificity: 96.61%; AUC: 0.590). The combination of *mSEPT9*, CEA, and CA19-9 further improved sensitivity, specificity, and AUC value (sensitivity: 78.43%; specificity: 86.07%; AUC: 0.878), respectively. Notably, the *mSEPT9* positivity rate was significantly associated with TNM stage, T stage, N stage, tumor size, vascular invasion, and nerve invasion among patients with CRC. A 100% correlation was observed between the positive results of the *mSEPT9* test and recurrence or metastasis in patients after therapeutic intervention.

**Conclusion:**

Our findings suggest that *mSEPT9* may represent a potential biomarker for the diagnosis and prognosis of CRC compared with CEA and CA19-9. Postoperative *mSEPT9* status may represent the first noninvasive marker of CRC recurrence or metastasis.

## 1. Introduction

Colorectal cancer (CRC) is the third-most common malignancy worldwide, with more than 1,800,000 diagnosed CRC cases and 881,000 deaths reported in 2018 [1]. In China, the incidence and mortality rates of CRC both rank fifth among those of all malignant cancers [2]; the incidence and mortality rates of CRC are predicted to increase along with the development of economy and the Westernization of lifestyle. Therefore, early diagnosis of CRC is crucial to improve patient outcomes. Currently, several conventional methods, such as the fecal occult blood test, colonoscopy, and computed tomography (CT) tests, are available to diagnose CRC; however, these approaches contain several limitations, such as low sensitivity, low specificity, invasiveness, or high cost, which restrict their clinical application [3–6]. In contrast, surgical approach represents the best treatment option for patients with CRC; however, recurrence or persistence after resection is associated with severe prognosis [7]. Approximately 30–50% of patients who undergo curative resection of CRC show CRC recurrence or metastasis [8]. An optimal surveillance protocol for CRC includes CT scans, colonoscopy, and serum carcinoembryonic antigen (CEA) level measurement, which has low sensitivity and specificity. Therefore, the development of patient-friendly and less invasive approaches with high sensitivity and specificity is imperative to improve patient outcomes.

Septin 9 DNA which encodes GTP-binding proteins plays an important role in the occurrence and progression of CRC [9]. Methylated septin 9 DNA (*mSEPT9*) has been detected in almost all CRC tissues [10]. Recently, studies have shown that *mSEPT9* represents a promising biomarker for CRC detection. Studies have shown that the rate of *SEPT9* methylation in peripheral blood of patients with CRC is related to clinicopathological features; for example, *SEPT9* methylation is positively correlated with the malignancy of CRC [11–13]. After radical resection of colorectal cancer, the level of *mSEPT9* in peripheral blood decreases; however, the level increases after recurrence, suggesting that *mSEPT9* in peripheral blood can be used for evaluating the pathological stages of CRC and may represent a molecular biological indicator for evaluating prognosis, recurrence, and metastasis [14, 15]. However, the reported sensitivity and specificity values of plasma *mSEPT9* are highly variable across studies, with the sensitivity ranging from 50.9–93.1% and specificity ranging from 62.2–93.8% [16, 17]. This may be attributed to the relatively small sample size. For example, the patient sample sizes in the studies were all less than 300, which may be insufficient for accurately assessing the prognostic value of *mSEPT9* in CRC.

In this study, we measured the cycle threshold (Ct) value of *mSEPT9* in 616 patients with CRC to analyze the value of *mSEPT9* in the diagnosis of CRC compared with CEA and carbohydrate antigen 19-9 (CA19-9). Second, we evaluated whether *mSEPT9* may play a potential role as a prognostic biomarker in CRC by evaluating the association between the positivity rate of *mSEPT9* and clinicopathological characteristics among patients with CRC. Furthermore, we analyzed the association between *mSEPT9* status and recurrence or metastasis in CRC. This study provides valuable information for the screening, diagnosis, and monitoring of CRC, especially in those patients who are reluctant to undergo colonoscopy or in cases where it is difficult to obtain biopsy specimens.

## 2. Material and Methods

### 2.1. Study Subjects and Samples

All samples were collected from Fujian Medical University Union Hospital, Fuzhou, China. Subjects were recruited between October 1, 2019, and May 31, 2021. The main inclusion criteria were adults (age, >18 years) with complete clinicopathological information and confirmed diagnosis of CRC (for the patient cohort) or no evidence of disease (NED, for the control group) based on imaging examination (including ultrasound, endoscopy, CT, and magnetic resonance imaging (MRI)) and/or subsequent pathological examination. Information on patient sex, age, and tumor/lymph node/metastasis (TNM) staging according to the American Joint Committee on Cancer TNM classification guidelines [18], primary tumor size, tumor location, cancer differentiation, vascular invasion, and nerve invasion were collected. The main exclusion criteria were history of any cancer, pregnancy, and incomplete information. Only subjects who underwent simultaneous evaluation of *mSEPT9*, CEA, and CA19-9 before any intervention were enrolled. Ultimately, 738 subjects were included in this study, including 616 patients with CRC (397 men and 219 women, median age (interquartile range, IQR): 61 (52–69) years) and 122 NED cases (69 men and 53 women, median age (IQR): 61 (52–69) years). This study was approved by the Research Ethics Committee at Fujian Medical University Union Hospital (Clinical trial registration number: 2021KJCX013). The participants provided informed consent for the collection of samples and clinicopathological information.

To evaluate the potential of *mSEPT9* to monitor recurrence/metastasis in CRC, we enrolled 18 patients who were either recently diagnosed and underwent initial treatment or were monitored for CRC recurrence/metastasis. Follow-up information, including the date of surgery, adjuvant treatment strategy, and recurrence status, were collected. Recurrences or metastases were determined based on diagnostic tests (colonoscopy, CT scans, MRI, or positron emission tomography scans) and confirmed via tissue pathology when available [19].

### 2.2. Plasma Preparation and Storage

Blood samples (10 mL) of each patient were collected in K2EDTA tubes (BD biosciences, Franklin Lake, NJ, USA) and processed immediately (<1 h) via double centrifugation at 1,400 × *g* for 12 min. The plasma obtained was transferred to a new tube and directly stored at -80°C for subsequent testing.

### 2.3. Analysis of the Methylation Status of Circulating SEPT9 DNA in Plasma

DNA was extracted from the plasma samples using a plasma processing kit (BioChain Science and Technology Inc., Beijing, China). The DNA sample was then incubated with bisulfite, during which unmethylated cytosine was converted to uracil, whereas methylated cytosine was not. Subsequently, the methylated target sequences in the bisulfite-converted DNA template were amplified via real-time polymerase chain reaction (PCR). PCR-blocking oligonucleotides and methylation-specific probes were used to distinguish between methylated and unmethylated DNA. PCR was performed in a 60 *μ*L reaction system. The thermocycling program was as follows: activation at 94°C for 20 min; 45 cycles at 62°C for 5 s, 55.5°C for 35 s, and 93°C for 30 s; and cooling at 40°C for 5 s. The methylation of *SEPT9* in plasma was measured using an ABI7500 fluorescent PCR instrument (Thermo Fisher Scientific, Waltham, MA, USA). Quantitative PCR was performed in duplicate, and the average Ct value was calculated. We recorded PCR data and then analyzed the *mSEPT9* and *β*-actin gene (*ACTB*) Ct within 45 cycles of amplification [7]. Results were considered valid when the *ACTB* Ct was ≤32.1, and the external negative and positive controls met the validity criteria specified by the manufacturer. An *mSEPT9* Ct value of ≤41 cycles was considered a positive result, while Ct > 41 or an undetermined Ct was considered a negative result (Table 1).

### 2.4. CEA and CA19-9 Levels

A total of 3–5 mL of venous blood was collected. Serum was isolated via centrifugation at 3,000 rpm for 10 min. The serum levels of CEA and CA19-9 were determined using a Cobas6000 Analyzer (Roche Diagnostics, Mannheim, Germany). The cut-off value for normal CEA was <5 ng/mL and that for normal CA19-9 was <37 U/mL, according to the manufacturer's instructions.

### 2.5. Statistical Analysis

Statistical analysis was performed using SPSS version 21.0 software (IBM, Armonk, NY, USA) or GraphPad Prism version 5.0 (GraphPad Software, San Diego, CA, USA). The diagnostic values of *mSEPT9*, CEA, and CA19-9 were estimated using receiver operating characteristic (ROC) curves. The Youden index was used to determine the optimal cut-off value to differentiate between healthy controls and patients with CRC. Combination analysis was performed using binary logistic regression. The relationship between *mSEPT9* and clinicopathological characteristics was assessed via a chi-square test. All tests were two-tailed, and a *p* value < 0.05 was considered significant.

## 3. Results

### 3.1. Performance of the mSEPT9 Assay for Detecting CRC

**Figure 1:**
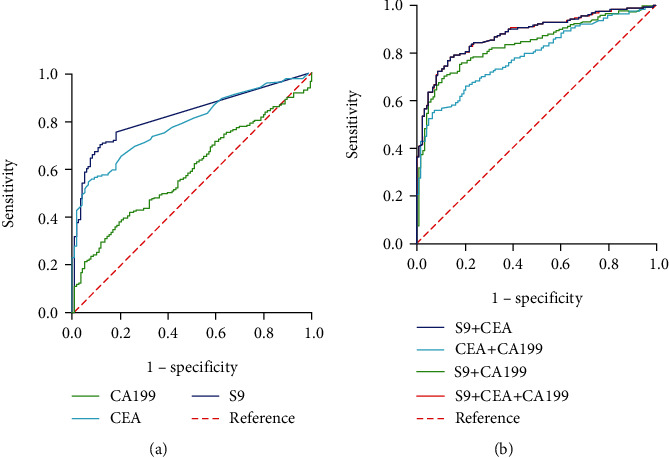
ROC curves of single *mSEPT9* (S9), CEA, CA19-9, and their combination in discriminating patients with CRC from healthy participants. (a) ROC curves of single S9, CEA, and CA19-9 in discriminating patients with CRC from healthy participants. (b) ROC curves of S9 + CEA, S9 + CA19 − 9, CEA + CA19 − 9, and S9 + CEA + CA19 − 9 in discriminating patients with CRC from healthy participants. ROC: receiver operating characteristic curve; *mSEPT9*: methylated septin 9; CEA: carcinoembryonic antigen; CA19-9: carbohydrate antigen-19-9.

To evaluate the performance of the blood *mSEPT9* assay in the diagnosis of CRC, we plotted ROC curves for cancer against NED compared with CEA and CA19-9, which are the most commonly used blood-based tumor markers in the diagnosis of CRC. The area under the ROC curve (AUC) for *mSEPT9*, CEA, and CA19-9 as parameters in the diagnosis of CRC was 0.826, 0.789, and 0.590, respectively (Figure 1 and Table 2). At the cut-off value of 41.0 for *mSEPT9*, we distinguished patients with CRC from healthy controls with a sensitivity of 72.94% and a specificity of 81.97% (Table 3). Notably, the diagnostic performance of CEA and CA19-9 could be improved when *mSEPT9*, CEA, and CA19-9 were combined for detection (Table 2). The AUCs for *mSEPT*9 + CEA and *mSEPT*9 + CA19 − 9 were 0.877 and 0.836, respectively (Figure 1 and Table 2), which were significantly higher than that for CEA + CA19 − 9 (AUC: 0.788). Moreover, the sensitivity of *mSEPT*9 + CEA was significantly higher than that of *mSEPT*9 + CA19 − 9 and CEA + CA19 − 9. When the three markers were combined, the AUC, sensitivity, and specificity of *mSEPT*9 + CEA + CA19 − 9 were 0.878, 78.43%, and 86.07%, respectively. Taken together, these results suggested that circulating *mSEPT9* may represent a promising biomarker for CRC. The detection of *mSEPT9*, CEA, and CA19-9 may provide better diagnostic performance in discriminating patients with CRC from healthy individuals, with higher sensitivity and specificity.

### 3.2. Association between Plasma mSEPT9 Status and Clinicopathological Characteristics of Patients with CRC

After determining the performance of the plasma *mSEPT9* assay for evaluating CRC, we further explored the correlation between *mSEPT9* status and clinicopathological characteristics. The positivity rate of *mSEPT9* was significantly higher in patients with more advanced TNM stages (stage I: 49.5%, stage II: 75.3%, stage III: 73.0%, stage IV: 81.8%, *p* = 0.0001) than that in patients with less advanced stages. Further analysis showed that *mSEPT9* positivity was also significantly greater in patients with a more advanced T stage (stage T1: 39.5%, stage T2: 51.4%, stage T3: 76.3%, stage T4: 77.2%, *p* = 0.0001) and N stage (stage N0: 67.0%, stage N1: 67.9%, stage N2: 80.3%, *p* = 0.004) than that in patients with less advanced stages, although there was no significant relationship between *mSEPT9* status and M stage (*p* = 0.220). The positivity rate of *mSEPT9* was also higher in CRC cases with vascular invasion (*p* = 0.007) and nerve invasion (*p* = 0.030) compared to those without (Table 3). Notably, patients with CRC containing a tumor size ≥ 5 cm showed a significantly higher positivity rate of *mSEPT9* than those with a tumor size < 5 cm (79.2% versus 65.5%, *p* = 0.0001). However, no significant association was found between *mSEPT9* positivity rate and gender, age, location, histological grade, CEA level, and CA19-9 level. Taken together, these data indicate that the methylation status of circulating *SEPT9* correlates with more advanced clinicopathological status in patients with CRC.

### 3.3. Plasma mSEPT9 Status for Monitoring CRC Recurrence

After validating the clinical value of *mSEPT9* for CRC diagnosis, we further investigated whether *mSEPT9* can be used as an indicator for recurrence or metastasis. We analyzed 18 CRC cases that were either recently diagnosed and underwent initial treatment or had been monitored for CRC recurrence (Table 4). The median period from primary diagnosis and treatment to *mSEPT9* measurement was 18 months, ranging from 6–28 months. In total, 6 of the 18 CRC cases showed recurrence or metastasis based on diagnosis, and all cases showed positive *mSEPT9* around the time of recurrence diagnosis. Notably, case no. 11 showed positive *mSEPT9* after curative surgery and chemotherapy (Table 4). In comparison, 4 of the 6 recurrent cases (66.7%) showed excessive CEA levels. No evidence of recurrence was found in the remaining 12 cases, which was consistent with the correspondingly negative *mSEPT9* status. Notably, in 11 of the remaining 12 cases, *mSEPT9* was positive before initial treatment, suggesting that besides monitoring CRC recurrence, *mSEPT9* may also be used for evaluating therapeutic efficacy in CRC.

## 4. Discussion

Early screening of CRC and efficient monitoring of metastases are urgently needed to improve the treatment outcomes of patients with CRC and reduce mortality. In this study, we evaluated the diagnostic value of *mSEPT9* in blood-based CRC detection in Chinese patients compared to that of two traditional blood-based tumor biomarkers (CEA and CA19-9). *MSEPT9* showed better performance than that of CEA and CA19-9 for CRC diagnosis, in which patients with CRC were distinguished from healthy individuals with a sensitivity of 72.94%, specificity of 81.97%, and AUC of 0.826. The combination of *mSEPT9* with CEA and CA19-9 further improved the sensitivity, specificity, and AUC value. Our statistical analysis also indicated that plasma *mSEPT9* DNA levels in patients with CRC were correlated with TNM stage, T stage, N stage, tumor size, vascular invasion, and nerve invasion. Furthermore, our data demonstrated that plasma *mSEPT9* may represent a reliable prognostic marker to predict recurrence or metastasis in patients with CRC, as well as in the evaluation of the therapeutic efficacy of multimodality therapy in CRC.

CRC represents a leading cause of cancer-related deaths worldwide, and patients with late-stage CRC have low five-year survival rates [2]. Blood-based screening strategies present the advantage of minimal invasiveness compared to that of endoscopy, and they are expected to have higher compliance rates than those for stool-based tests [20]. However, the sensitivity and specificity of current blood-based markers, CEA and CA19-9, have been demonstrated to be low, especially for stratifying early stages of CRC [21, 22], which was further confirmed in our study. Although invasive colonoscopy has the highest sensitivity and specificity for CRC, it has the lowest patient compliance rate due to the need of bowel preparation and discomfort during the test. Furthermore, certain patients with severe cardiopulmonary insufficiency, enterostenosis, or intestinal perforation cannot undergo invasive tests. Hence, novel markers are sought after to improve the sensitivity and specificity in screening for CRC in patients [23]. Our data showed that plasma *mSEPT9* had an AUC of 0.826 (95% confidence interval: 79.15–86%) for CRC detection with high sensitivity and specificity, which is similar to that reported previously [17]. Our findings also showed that the combined detection of *mSEPT9*, CEA, and CA19-9 improved the diagnostic performance of CEA and CA19-9 in discriminating patients with CRC from healthy participants. Taken together, plasma *mSEPT9* represents a potential blood-based biomarker for the diagnosis of CRC, and this blood-based test is noninvasive, patient friendly, and is expected to obtain high compliance.

To the best of our knowledge, few studies have investigated the association between *mSEPT9* status and clinicopathological characteristics of patients with CRC. TNM stage, which is based on the extent of tumor growth (T), the extent of spread to the lymph nodes (N), and the presence of metastasis (M), is the most studied classification system for evaluating CRC. The positive rates of *mSEPT9* were significantly associated with the TNM stage, including T and N stages, and these findings were consistent with those of Sun et al. [24]. However, a previous study by Fu et al. [17] on 98 CRC cases showed no significant association between *mSEPT9* and TNM stage, which may be caused by a relatively smaller sample size. Previous studies showed that vascular invasion and lymph node metastases are negatively associated with prognosis and represent potential independent prognostic markers of CRC [25–27]. However, few accurate protein biomarkers of vascular invasion in CRC are available. Our results showed that the positive rate of *mSEPT9* was significantly associated with vascular invasion and nerve invasion. Notably, our data demonstrated that the positive rate of *mSEPT9* in CRC cases with a tumor size of ≥5 cm was significantly higher than that in cases with a tumor size of <5 cm, which was similar to the findings of Fu et al. [17]. We speculate that the status of *mSEPT9* has a positive correlation with tumor size. These findings also suggested that plasma *mSEPT9* has potential as an additional biomarker for prognostic evaluation of CRC.

It is important to detect CRC recurrence or metastasis in postoperative patients. To our knowledge, CEA is the only blood-based test applied for conventionally monitoring CRC recurrences; however, it has low sensitivity and specificity [19, 28]. Our data showed a good agreement between the *mSEPT9* status and CRC recurrence (100% sensitivity), suggesting that the *mSEPT9* test may represent a reliable marker in monitoring CRC recurrence or metastasis. Additionally, our results revealed for the first time that the persistent positivity of plasma *mSEPT9* after multimodality therapy was highly correlated with impending recurrence or metastasis, whereas the conversion of *mSEPT9* positivity to negativity indicated the end of recurrence. Based on this perspective, *mSEPT9* may serve as a reliable biomarker for assessing therapeutic efficacy in patients with CRC whose preoperative *mSEPT9* was positive. Since this is a pilot study, we will further verify the potential predictive ability of *mSEPT9* for tumor recurrence/metastasis by extending follow-up time, *mSEPT9* monitoring, and combining samples from multiple medical centers to expand the sample size.

Some limitations of this study need to be addressed. First, the short follow-up duration in this study with single-centered retrospective design may provide bias towards sample selection and analysis. Moreover, we failed to collect data on overall survival; therefore, the relationship of *mSEPT9* status with overall survival in patients with CRC was not evaluated. Future studies should further confirm whether plasma *mSEPT9* has the potential to provide clinically relevant lead times compared to that of conventional diagnostic modalities for recurrence or metastasis detection, as well as in assessing the therapeutic efficacy in CRC. Hence, further prospective multicenter studies are needed to validate the clinical significance of *mSEPT9* in patients with CRC.

## 5. Conclusion

In summary, our results showed that plasma *mSEPT9* represents a promising biomarker in CRC diagnosis. Notably, we revealed a significant association between *mSEPT9* status and clinicopathological characteristics of patients with CRC. Postoperative *mSEPT9* during follow-up served as a significant indicator for CRC recurrence or metastasis.

## Figures and Tables

**Table 1:** Criteria for the validity of the system according to manufacturer's instructions.

Septin 9 result	*mSEPT9*	*ACTB*
Positive	Ct ≤ 41.0	Ct ≤ 32.1
Negative	Undetermined or Ct > 41.0	Ct ≤ 32.1
Invalid	Any case	Ct>32.1

*mSEPT9*: methylated septin 9 DNA; *ACTB*: *β*-actin; Ct: threshold amplification cycle.

**Table 2:** The values of S9, CEA, and CA19-9 alone and in combination for differential diagnosis of health donors and patients with CRC.

Variable	AUC	Cut-off	Sensitivity	Specificity	95% confidence interval	
					Upper limit	Lower limit
S9	0.826	41	72.94%	81.97%	79.15%	86.00%
CEA	0.789	5	43.96%	96.72%	75.13%	82.59%
CA19-9	0.590	37	14.99%	96.61%	53.89%	64.05%
S9 + CEA	0.877		78.43%	85.25%	84.88%	90.61%
S9 + CA19 − 9	0.836		66.91%	91.80%	80.27%	87.01%
CEA + CA19 − 9	0.788		55.76%	92.62%	75.04%	82.53%
S9 + CEA + CA19 − 9	0.878		78.43%	86.07%	84.89%	90.62%

S9: methylated septin 9 DNA; CEA: carcinoembryonic antigen; CA19-9: carbohydrate antigen-19-9; CRC: colorectal cancer.

**Table 3:** Relationship between *mSEPT9* and pathological characteristics of patients with CRC.

Variables	Total	S9-positive cases	S9-negative cases	*p* value
CRC cases	616	440 (71.4%)	176 (28.6%)	
Gender				0.079
Male	397	293 (73.8%)	104 (26.2%)	
Female	219	147 (67.1%)	72 (32.9%)	
Age				0.137
<60	272	186 (68.4%)	86 (31.6%)	
≥60	344	254 (73.8%)	90 (26.2)	
Location				0.664
Colon	303	221 (72.9%)	82 (27.1%)	
Rectosigmoid transition	12	9 (75%)	3 (25%)	
Rectum	301	210 (69.8%)	91 (30.29%)	
TNM stage				**0.0001**
I	91	45 (49.5%)	46 (50.5%)	
II	170	128 (75.3%)	42 (24.7%)	
III	267	195 (73.0%)	72 (27.0%)	
IV	88	72 (81.8%)	16 (18.2%)	
T stage				**0.0001**
T1	38	15 (39.5%)	23 (60.5%)	
T2	70	36 (51.4%)	34 (48.6%)	
T3	350	267 (76.3%)	83 (23.7%)	
T4	158	122 (77.2%)	36 (22.8%)	
N stage				**0.004**
N0	264	177 (67.0%)	87 (33.0%)	
N1	156	108 (67.9%)	51 (32.1%)	
N2	193	155 (80.3%)	38 (19.7%)	
M stage				0.220
M0	528	382 (72.3%)	146 (27.7%)	
M1	88	58 (65.9%)	30 (34.1%)	
Histological grade				0.836
Low	42	30 (71.4%)	12 (28.6%)	
Moderate	560	401 (71.6%)	159 (28.4%)	
High	14	9 (64.3%)	5 (35.7%)	
Vascular invasion				**0.007**
Absent	89	59 (66.3%)	30 (33.7%)	
Present	410	309 (75.4%)	101 (24.6%)	
Unknown	117	72 (61.5%)	45 (38.5%)	
Nerve invasion				**0.030**
Absent	133	97 (72.9%)	36 (27.1%)	
Present	366	271 (74.0%)	95 (26.0%)	
Unknown	117	72 (61.5%)	45 (38.5%)	
Tumor size (cm)				**0.0001**
<5	351	230 (65.5%)	121 (34.5%)	
≥5	265	210 (79.2%)	55 (20.8%)	
CEA				0.061
<5	341	254 (74.5%)	87 (25.5%)	
≥5	275	186 (67.6%)	89 (32.4%)	
CA19-9				0.576
<37	519	373 (71.9%)	146 (28.1%)	
≥37	97	67 (69.1%)	30 (30.9%)	

*S9*: methylated septin 9 DNA; CEA: carcinoembryonic antigen; CA19-9: carbohydrate antigen-19-9; CRC: colorectal cancer.

**Table 4:** Detection of CRC recurrence based on plasma *mSEPT9* during follow-up.

No.	Gender	Age (years)	TNM staging	Treat	Period^†^ (months)	S9		CEA (ng/mL)	Recurrence status
						Pre	Pos		
1	Male	60	T3N0M0	S	26	+	-	3.1	NER
2	Male	56	T4aN2M0	S + C	26	+	+	**153.4**	Retroperitoneal lymph node metastases
3	Female	59	T4bN2M0	S + C	20	+	+	**50.2**	Lung metastases
4	Male	55	T4aN2M0	S + C + R	18	+	-	1.8	NER
5	Male	61	T4bN2M0	S + C	18	+	-	3.0	NER
6	Male	57	T4aN2M0	S + C	15	+	+	4.6	Liver metastases
7	Male	38	T4bN1M0	S + C + R	16	-	-	1.4	NER
8	Female	70	T2N0M0	S	18	+	-	3.0	NER
9	Female	49	T3N2M0	S + R	18	+	-	1.9	NER
10	Female	61	T4bN1M0	S + C + R	18	+	+	**34.4**	Liver metastases
11	Male	50	T4bN1M0	S + C	18	-	+	**24.0**	Liver metastases
12	Male	51	T3N0M0	S + C	18	+	-	1.6	NER
13	Female	64	T3N0M0	S + C	18	+	-	1.2	NER
14	Female	69	T4N2M0	S + C	6	+	-	1.7	NER
15	Female	51	T3N2M0	S + C	18	+	-	1.1	NER
16	Male	47	T4N2M0	S + C	17	+	-	3.6	NER
17	Male	51	T2N0M0	S	28	+	-	2.0	NER
18	Female	78	T4N0M0	S + C	6	+	+	1.5	Recurrent CRC

*S9*: methylated septin 9 DNA; Treat: treatment; S: curatively intended surgery; C: chemotherapy; R: radiation therapy; NER: no evidence of recurrence; +: positive; −: negative; boldface in CEA column represents positive; †: period after treatment.

## Data Availability

The data used to support the findings of this study are included within the article.
